# Angular Misalignment Calibration for Dual-Antenna GNSS/IMU Navigation Sensor

**DOI:** 10.3390/s23010077

**Published:** 2022-12-21

**Authors:** Alexander Kozlov, Fedor Kapralov

**Affiliations:** Faculty of Mechanics and Mathematics, Lomonosov Moscow State University, 119991 Moscow, Russia

**Keywords:** inertial sensors, multi-antenna GNSS, angular misalignment, calibration

## Abstract

We address the angular misalignment calibration problem, which arises when a multi-antenna GNSS serves as a source of aiding information for inertial sensors in an integrated navigation system. Antennas usually occupy some outside structure of the moving carrier object, whilst an inertial measurement unit typically remains inside. Especially when using low- or mid-grade MEMS gyroscopes and accelerometers, it is either impossible or impractical to physically align IMU-sensitive axes and GNSS antenna baselines within some 1–3 degrees due to the micromechanical nature of the inertial sensors: they are just too small to have any physical reference features to align to. However, in some applications, it is desirable to line up all sensors within a fraction-of-a-degree level of accuracy. One may imagine solving this problem via the long-term averaging of sensor signals in different positions to ensure observability and then using angle differences for analytical compensation. We suggest faster calibration in special rotations using sensor fusion. Apart from quicker convergence, this method also accounts for run-to-run inertial sensor bias instability. In addition, it allows further on-the-fly finer calibration in the background when the navigation system performs its regular operation, and carrier objects may undergo gradual deformations of its structure over the years.

## 1. Introduction

One of the key problems in using low-grade inertial measurement units (IMU) is their inability to perceive azimuth without external aids. High-grade strapdown inertial navigation systems (INS) do this by measuring the Earth’s rotation rate components in their instrumental axes. Apart from direct vector matching, there exist a number of approaches [1], but the crux remains in the accuracy of angular rate sensors (gyroscopes) being well within a small fraction of the Earth’s angular rate magnitude. For microelectromechanical sensors (MEMS), run-to-run bias instability typically exceeds this requirement by 1–3 orders of magnitude, making conventional azimuth perception virtually impossible. For sensors having better in-run stability, special gyrocompassing methods have been developed [2], which require some special rotation of the IMU and a decent time span. In some cases, these methods highly rely on the physical stability of the base, so that the slightest mechanical perturbation may entirely ruin the solution. Even conventional sensor fusion with a single-antenna global navigation satellite system (GNSS) has its capabilities quite limited in estimating azimuth attitude error for low-grade gyroscopes.

**Figure 1:**
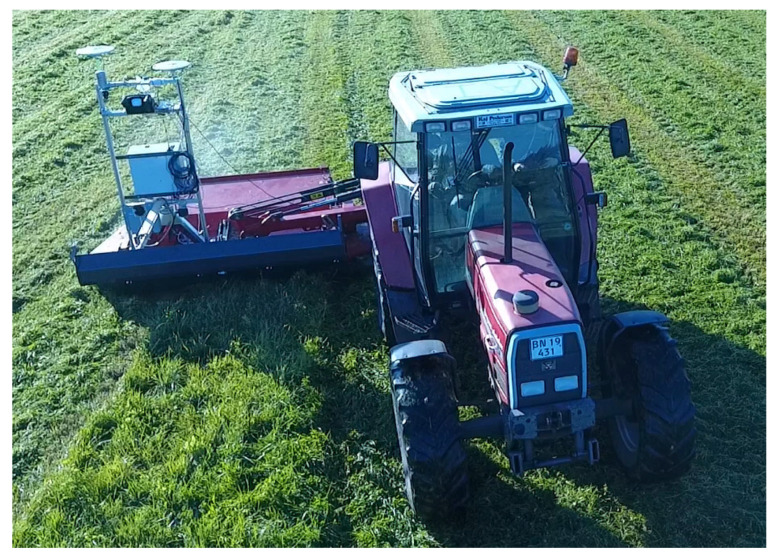
An example of a dual-antenna GNSS setup (see top left corner) mounted on a vehicle with the baseline lying sideways from one antenna to another. Image courtesy of Kragh et al. [3].

To tackle this issue, a dual-antenna GNSS setup has become rather popular in a variety of applications [3,4,5,6,7,8] since cheaper and more compact GNSS hardware had emerged on the market. Its baseline vector, i.e., the vector connecting phase centers of the antennas (see Figure 1), being both known in the body reference frame of a vehicle and measured in a navigation reference (say, East–North–Up axes) using GNSS carrier phase observables and an RTK (real-time kinematics) approach [9], yields two attitude angles almost instantly as compared to MEMS gyrocompassing with no special maneuvering necessary. If the baseline lies sideways relative to the carrier, it provides yaw and roll angles of the body, with pitch, i.e., the rotation angle around the baseline itself, remaining unknown. Still, the inertial sensor provides pitch orientation, so that a full attitude solution becomes available in this integrated sensor system.

However, some use cases require a sub-degree level of attitude accuracy, which implies both dual-antenna GNSS and inertial systems well aligned within the vehicle’s frame of reference. For GNSS antennas with proper phase center calibration, their locations usually match the intended positions in technical drawings. The latter generally have millimeter-level precision, so that corresponding angular deviations on a 1 m baseline become small fractions of a degree. They do less well with the inertial sensors. Due to the size of MEMS gyroscopes and accelerometers being extremely small, they barely have physical features to align them better than within some 3. Therefore, after installation, the IMU module has some unknown angular misalignment with respect to GNSS antennas.

Due to its rather niche application, only a few works address the above issue [4,6,10]. We have been unable to find published methods, which still may exist in the Web. From personal communication, it appears that angular misalignment calibration either requires a special static experiment over a long period of time when both attitude solutions are averaged, or it emerges from the manual analysis of deviations in the integrated attitude solution. While the first solution relies upon inertial sensor biases being stable enough and takes a lot of time, the drawbacks of the second approach are self-evident.

In this paper, we suggest a solution to the misalignment problem via its calibration based on sensor fusion algorithms in a special experiment. Apart from faster convergence, this method accounts for run-to-run inertial sensor bias instability. In addition, it allows further on-the fly finer calibration in the background when a navigation system performs its regular operation, and the carrier object may undergo gradual deformations of its structure over the years.

While our calibration method is based on conventional Kalman filtering and INS error equations, we have identified four key issues that appear to be essential to solving the problem. They have not been self-apparent prior to approaching the problem, and therefore, we would like to emphasize these issues for those who may be working on similar problems:

- Ensuring observability and good estimability in the underlying estimation problem requires specific rather intensive rotations;
- For those above-mentioned rotations, attitude integration algorithms must provide a numerical solution which conforms to the INS error equations closely enough; for example, simplest single-step Euler integration does not do so;
- Timing delays between GNSS and IMU measurements should be a part of the estimation problem, since their magnitude even of a fraction of an IMU time step is significant enough;
- In addition, gyroscope measurements should be extrapolated accordingly when transforming GNSS antenna velocity into the velocity of the IMU.

**Figure 2:**
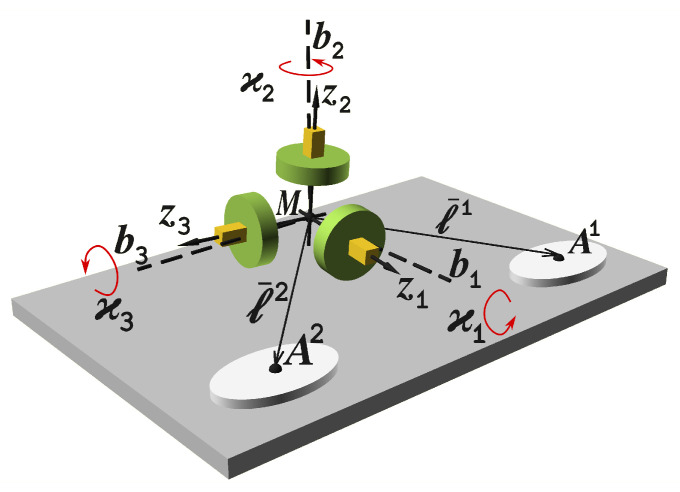
Sensor setup. Two antennas ($A^{1}$ and $A^{2}$) of a dual-antenna GNSS unit reside at locations given by lever arms ${\bar{\ell }}^{1}$ and ${\bar{\ell }}^{2}$ relative to the inertial measurement unit with its reference point *M*. IMU instrumental axes $z_{1}$, $z_{2}$ and $z_{3}$ are fixed to inertial sensor array, while lever arm vectors are known in a carrier body reference frame. Three components $\varkappa_{1}$, $\varkappa_{2}$ and $\varkappa_{3}$ of the Euler rotation vector define the slight misalignment between the two reference frames.

In the following sections, we formulate misalignment calibration as an optimal estimation problem for a dynamic system with measurements. Its mechanization equations are based on INS error equations, with additional parameters being modeled as constants. For the complete rationale and derivation, please see the sections below. We consider the following sensor setup shown in Figure 2 with the notation listed in Table 1.

We assume every quantity as a function of time, so that *t* may appear as its argument, if necessary. However, in most cases, it is omitted for brevity.

Each vector symbol may have a subscript letter denoting either a reference frame in which its components are considered (e.g., *x*, *b*, *z*), or a specific axis (E, N, U, etc.) The dot above any quantity [x]˙ represents its derivative over time. For the vector product with any vector $\bar{v}$, we introduce a linear operator $\hat{v}$ so that in its coordinate form, the vector product becomes multiplication by a matrix:

v^=−0−v3−v2−v3−0−v1−v2−v1−0,v^[·]=def[·]×v

Let *I* be the identity matrix of the appropriate size whenever it is being used. We consider all reference frames to be right-handed orthogonal, so that the IMU has been calibrated with sufficient accuracy. The relation between attitude angles and attitude matrix is as follows:

(1) L(γ,θ,ψ)=[−cosθsinψ−cosθcosψ−sinθ−sinθsinψcosγ+cosψsinγ−sinθcosψcosγ−sinψsinγ−cosθcosγ−sinθsinψsinγ+cosψcosγ−sinθcosψsinγ−sinψcosγ−cosθsinγ],

## 2. Theoretical Background

### 2.1. Inertial Solution

To formulate the INS error equations, one must obtain the inertial solution first. It uses measurements from inertial sensors—angular rate sensors (gyroscopes) and accelerometers—for integrating equations of motion and yields attitude, position and velocity solution over time. The equations may appear in different form, and here, we use the attitude matrix and geodetic coordinates to use the same equations not only for calibration but in INS regular operation as well. They are as follows:

(2) $$\begin{array}{ccc} \dot{\lambda \;\;} & = & \frac{V{}_{E}}{(R{}_{E}+h)\cos\varphi },\;\dot{\varphi }=\frac{V{}_{N}}{R{}_{N}+h},\;\dot{h\;\;}=V{}_{U},\\ \dot{V_{x}\;} & = & \left({\hat{\Omega }}_{x}+2{\hat{u}}_{x}\right)V_{x}+g_{x}+L{}^{T}f_{z},\\ \dot{L\;\;} & = & {\hat{\omega }}_{z}L-L\left({\hat{\Omega }}_{x}+{\hat{u}}_{x}\right), \end{array}$$

where $g_{x}$ includes the centrifugal specific force component, and Ω is an angular velocity of the navigation reference frame Mx relative to the Earth, with its components being:

(3) $$\Omega_{x}=\left[\begin{array}{ccc} -\frac{V{}_{N}}{R{}_{N}+h} & \frac{V{}_{E}}{R{}_{E}+h} & \frac{V{}_{E}\mathrm{tg}\varphi }{R{}_{E}+h} \end{array}\right]{}^{T}\;\;\;\;,\;R{}_{E}=\frac{a}{\sqrt{1-e^{2}\sin^{2}\varphi }},\;R{}_{N}=R{}_{E}\frac{1-e^{2}}{1-e^{2}\sin^{2}\varphi },$$

with *a* and *e* being the Earth’s ellipsoid semimajor axis and eccentricity, respectively. The first three equations in (2) actually describe a radius vector $r_{x}$ in some Earth-centered Cartesian reference frame. These two variants may be used interchangeably.

It is common to omit some of the terms such as Coriolis acceleration or even the Earth’s rotation in the above equations for lower grade inertial units. In the following derivations we will, however, keep these terms for the pure sake of mathematical rigor. In real-world applications, the corresponding algorithms may happen to be previously implemented and tested in navigation software or libraries. Given that for modern processors, the additional computational burden often appears neglectable, we find it appropriate to leave for each reader the decision of whether to simplify the equations or not.

In addition, in real navigation systems, the equations for the altitude and vertical velocity component in (2) introduce well-known exponential instability [1]. So, instead of integrating them, the system uses an external source for altitude. In our misalignment calibration, however, we may benefit from using vertical velocity measurements from GNSS, like from using horizontal ones. We therefore keep these equations from being integrated and use them to form INS error equations as well.

The starting position and velocity in (2) are trivial to specify, with the coordinates of the calibration experiment known and velocity being zero. Attitude determination, however, requires a special procedure called *initial alignment*.

#### 2.1.1. Initial Alignment Procedure

In this section, we describe the procedure required to obtain initial estimates for the attitude matrix $L^{0}$. For doing that, the IMU remains at rest on the ground for some time $t_{0}$ with its normal axis pointing approximately upwards. While being stationary, its accelerometers measure the ground reaction force opposite to gravity acceleration, so that

(4) $$L{}^{T}f_{z}+g_{x}=0.$$

From the above, after averaging accelerometer outputs over the time period $t\in [0,t_{0}]$, one may estimate IMU roll and pitch angles, respectively, as

(5) $$\gamma^{\prime }\left(t_{0}\right)=\mathrm{arctg}\frac{\langle f_{z3}^{\prime }\rangle }{\langle f_{z2}^{\prime }\rangle },\;\theta^{\prime }\left(t_{0}\right)=\mathrm{arctg}\frac{\langle f_{z1}^{\prime }\rangle }{\sqrt{{\langle f_{z2}^{\prime }\rangle }^{2}+{\langle f_{z3}^{\prime }\rangle }^{2}}},$$

with the prime [x]′ symbol meaning a value derived from measurements, and angle brackets $\langle \cdot \rangle$ for averaging over $t\in [0,t_{0}]$. The two-antenna GNSS solution then provides an estimate $\psi^{\prime }\left(t_{0}\right)$ for the azimuth angle up to some misalignment and other errors. According to (1), the estimated initial attitude matrix becomes:

(6) $$L^{\prime }\left(t_{0}\right)=L\left(\gamma^{\prime }\left(t_{0}\right),\theta^{\prime }\left(t_{0}\right),\psi^{\prime }\left(t_{0}\right)\right).$$

In addition to obtaining the initial attitude matrix, it is usually makes sense for MEMS gyroscopes to obtain rough estimates for their in-run biases ${\tilde{\nu }}_{z1}^{0},\;{\tilde{\nu }}_{z2}^{0},\;{\tilde{\nu }}_{z3}^{0}$, since for most devices, they exceed tens of degrees per second, being greater than or comparable to the Earth’s angular rate of 15°/h. Given the IMU is stationary during the initial alignment, the simplest form for those estimates is:

(7) $$\left[\begin{array}{ccc} {\tilde{\nu }}_{z1}^{0} & {\tilde{\nu }}_{z2}^{0} & {\tilde{\nu }}_{z3}^{0} \end{array}\right]{}^{T}\overset{def}{=}{\tilde{\nu }}_{z}^{0}\left(t_{0}\right)=\langle \omega_{z}^{\prime }\rangle -L^{\prime }\left(t_{0}\right)u_{x}.$$

According to Section 2.1, there is one more feature to the initial alignment in our misalignment calibration experiment. Namely, we are going to integrate the equations of motion (2) along the vertical axis. To avoid introducing exponential instability into the solution, we use a constant gravity model for our misalignment experiment with a gravity acceleration value of

(8) $$g^{0}\overset{def}{=}∥\langle f_{z}^{\prime }\rangle ∥,\;\mathrm{so}\;\mathrm{that}\;g_{x}^{\prime }=\left[\begin{array}{ccc} 0 & 0 & -g^{0} \end{array}\right]{}^{T}\;\;.$$

The model (8) certainly has some constant bias $\Delta g{}_{U}$ produced by accelerometer errors. However, this bias appears to introduce no error into calibration, being properly estimated along with other parameters (see Section 2.4 for details).

#### 2.1.2. Attitude Integration

For low-grade strapdown inertial systems such as MEMS-based IMUs, one usually implements simpler versions of attitude integration algorithms such as the Euler method for quaternions [11]. However, our simulation has shown (see Section 3.4) that motion patterns which provide better estimability properties of the misalignment calibration should include some kind of conical rotation. Under such motion, those simpler methods tend to introduce significant numerical errors, which do not obey INS error equations. Being systematic, they in turn produce biased estimates in calibration. With that knowledge, we have decided to use a more accurate version of the attitude integration algorithm based on the Bortz kinematic equation [12] for a Euler rotation vector $\bar{\phi }$:

(9) $$\dot{\bar{\phi }}=\bar{\omega }+\frac{1}{2}\bar{\phi }\times \bar{\omega }+\frac{1}{∥\bar{\phi }{∥}^{2}}\left[1-\frac{∥\bar{\phi }∥\sin∥\bar{\phi }∥}{2\left(1-\cos∥\bar{\phi }∥\right)}\right]\;\cdot \bar{\phi }\times \left[\bar{\phi }\times \bar{\omega }\right],\;∥\bar{\phi }∥\neq \pi k,\;\;k\in Z.$$

For (9), we use an approximation of the 4-th order Runge–Kutta integration method. Although it may seem excessive to use it for a low-grade IMU, one should keep in mind that its errors are either systematic and closely conformant to INS error equations or stochastic with a nearly zero mean cumulative effect. Moreover, since we have used this algorithm for processing simulated data, it seems consistent to use it for real experiments as well. For the transition between two time instants $t_{i-1}$ and $t_{i}$ with time step Δt between them, we have:

(10) $$\begin{array}{ccc} F_{1} & = & F\left({\tilde{\omega }}_{z}\left(t_{i-1}\right),\;0\right),\;{\tilde{\omega }}_{z}\left(t_{i-1}\right)=\frac{\omega_{z}^{\prime }\left(t_{i}\right)+\omega_{z}^{\prime }\left(t_{i-1}\right)}{2},\\ F_{2} & = & F\left(\omega_{z}^{\prime }\left(t_{i}\right),\;F_{1}\frac{\Delta t}{2}\right),\;F_{3}=F\left(\omega_{z}^{\prime }\left(t_{i}\right),\;F_{2}\frac{\Delta t}{2}\right),\\ F_{4} & = & F\left({\tilde{\omega }}_{z}\left(t_{i}\right),\;F_{3}\Delta t\right),\;{\tilde{\omega }}_{z}\left(t_{i}\right)=\frac{3\omega_{z}^{\prime }\left(t_{i}\right)-\omega_{z}^{\prime }\left(t_{i-1}\right)}{2},\\ \Delta \bar{\phi } & = & \frac{F_{1}+2F_{2}+2F_{3}+F_{4}}{6}\Delta t,\\ F(\omega_{z},\;\phi_{z}) & \;\overset{def}{=}\; & \omega_{z}+\frac{1}{2}\phi_{z}\;\times \;\omega_{z}+\left(\frac{1}{12}+\frac{∥\phi_{z}{∥}^{2}}{720}\right)\phi_{z}\;\times \;\left(\phi_{z}\;\times \;\omega_{z}\right), \end{array}$$

where $\tilde{\omega }\left(t_{i-1}\right)$ and $\tilde{\omega }\left(t_{i}\right)$ approximate the instant rotation rate vector using gyroscope measurements, which are the average angular rate components over the respective time step. The function $F(\omega_{z},\phi_{z})$ is the fourth-order Taylor expansion of the right-hand part of the Bortz equation with $∥\phi_{z}∥\;\ll \;1$. The calculated Euler vector increment $\Delta \phi_{z}$ yields a transition matrix *C* via Euler–Rodrigues’ rotation formula [12] as follows:

(11) $$C=I+\frac{\sin∥\Delta \phi_{z}∥}{∥\Delta \phi_{z}∥}{\hat{\Delta \phi }}_{z}+\frac{1-\cos∥\Delta \phi_{z}∥}{∥\Delta \phi_{z}{∥}^{2}}{\hat{\Delta \phi }}_{z}^{2}.$$

Together with the transition matrix for the navigation frame using the regular Euler method, we perform mechanization for the attitude matrix *L* from a time instant $t_{i-1}$ to $t_{i}$:

(12) $$L\left(t_{i}\right)=C\;L\left(t_{i-1}\right)\;B{}^{T},\;B=I+\frac{\sin∥\epsilon_{x}∥}{∥\epsilon_{x}∥}{\hat{\epsilon }}_{x}+\frac{1-\cos∥\epsilon_{x}∥}{{∥\epsilon ∥}^{2}}{\hat{\epsilon }}_{x}^{2},\;\epsilon_{x}\;\overset{def}{=}\;\left(\Omega_{x}+u_{x}\right)\Delta t.$$

Using (12), we obtain a calculated attitude matrix $L^{\prime }$ over time, starting with $L^{\prime }\left(t_{0}\right)$ from the initial alignment procedure.

#### 2.1.3. Position and Velocity Integration

For the position and velocity, the conventional modified Euler integration has proven to work well, so that according to (2):

(13) rx′(ti)=rx′(ti−1)+Vx′(ti−1)Δt,Vx′(ti)=Vx′(ti−1)+Ω^x′+2u^x′Vx′+gx′t=ti−1Δt+L˜(ti−12)Tfz′(ti)Δt,L˜(ti−12)=defI+ωz′(ti)Δt2L′(ti−1),

with an appropriate gravity model for $g_{x}^{\prime }$, and L˜(ti−12) being an estimate for the mid-step attitude matrix. Our calibration experiment does not include active linear motion, so (13) may be simplified. The reason for not ignoring here the Coriolis term and the rotation of navigation frame ($\Omega_{x}$) is our future plan to use the same equations and models for in-run system calibration in its regular operation.

As for the gravity model $g_{x}^{\prime }$, we use a constant value obtained in the initial alignment, as per Section 2.1.1, for our nearly static calibration experiment to avoid exponential instability. In other cases, appropriate gravity models may be used for integration, which are provided with an external altitude information.

### 2.2. INS Instrumental Errors Model

In general, the choice of mathematical model of INS instrumental errors heavily relies on accuracy class of the INS. For us, the subject is a low- or mid-grade MEMS IMU. We assume that before calibrating the angular misalignment, the inertial sensors themselves are pre-calibrated, so that standard parameters of an INS instrumental errors model, i.e., constant biases, scaling coefficients, etc., are compensated using one of the known methods [13,14,15]. In addition, temperature variations of inertial sensor measurements are not considered in this research. We assume that IMU thermal calibration can be carried out in advance [16,17], and residual errors are stochastic. Otherwise, we may suggest performing the misalignment calibration at a constant temperature. Let us define accelerometers and gyroscopes instrumental errors as

(14) $$\begin{array}{c} \Delta f_{z}\overset{def}{=}f_{z}^{\prime }-f_{z},\;-\nu_{z}\overset{def}{=}\omega_{z}^{\prime }-\omega_{z}, \end{array}$$

where the minus sign in the second expression of (14) originates from a tradition for gimbaled INS. We accept the following model for instrumental errors of accelerometers and gyroscopes

(15) $$\begin{array}{c} \Delta f_{z}=\Delta f_{z}^{0}+\Delta f_{z}^{s},\;\nu_{z}=\nu_{z}^{0}+\nu_{z}^{s}, \end{array}$$

where $\Delta f_{z}^{0}$, $\nu_{z}^{0}$ are null biases of accelerometers and gyroscopes, respectively, $\Delta f_{z}^{s}$, $\nu_{z}^{s}$ are stochastic terms of the measurement error with known a priori moments. We include null biases into the estimation process since they generally happen to be different and not very stable in each INS run as opposed to scaling factors and other parameters. For their instability, we accept Wiener processes:

(16) $$\begin{array}{c} \Delta {\dot{f}}_{z}^{0}=q_{\Delta f^{0}},\;{\dot{\nu }}_{z}^{0}=q_{\nu^{0}}, \end{array}$$

where $q_{\Delta f^{0}}$, $q_{\nu^{0}}$ represent independent white noise processes with known a priori moments.

### 2.3. INS Error Equations

The INS errors in the geodetic navigation frame are as follows:

- $\Delta r_{x}=\left(\Delta r{}_{E},\;\Delta r{}_{N},\;\Delta r{}_{U}\right){}^{T}$ is the position error;
- $\delta V_{x}=(\delta V{}_{E}$, $\delta V{}_{N},\;\delta V{}_{U}){}^{T}$ is the velocity error;
- $\alpha {}_{E}$, $\alpha {}_{N}$ indicates the deflection of virtual horizon,
- $\beta {}_{U}$ is the azimuth attitude error.

We further consider the behaviour of INS errors over time up to linear terms. We use INS error equations [18] in the computed geodetic navigation frame *y* ($My_{1}y_{2}y_{3}$). The INS error equations will serve as a dynamic model in the linear estimation problem. We adapt these equations for the case of specific calibration motions, the choice of which will be explained in Section 3.4. The modification consists of replacing the term $2\omega_{0}^{2}\Delta r{}_{U}$ with $\omega_{0}$ being the Schuler frequency, by the constant error of local gravity force $\Delta g{}_{U}$ resulting from accelerometer errors in initial alignment as described in Section 2.1.1. Thus, we have:

(17) $$\begin{array}{ccc} \dot{\Delta r}{}_{E} & = & \delta V{}_{E}-V{}_{U}\alpha {}_{N}+V{}_{N}\beta {}_{U}+\frac{V{}_{U}}{R{}_{E}+h}\Delta r{}_{E}+\Omega {}_{U}\Delta r{}_{N}-\Omega {}_{N}\Delta r{}_{U},\\ \dot{\Delta r}{}_{N} & = & \delta V{}_{N}+V{}_{U}\alpha {}_{E}-V{}_{E}\beta {}_{U}+\frac{V{}_{U}}{R{}_{N}+h}\Delta r{}_{N}-\Omega {}_{U}\Delta r{}_{E}+\Omega {}_{E}\Delta r{}_{U},\\ \dot{\Delta r}{}_{U} & = & \delta V{}_{U}-V{}_{N}\alpha {}_{E}+V{}_{E}\alpha {}_{N},\\ \dot{\delta V}{}_{E} & = & +\left(2u{}_{U}+\Omega {}_{U}\right)\delta V{}_{N}-\left(2u{}_{N}+\Omega {}_{N}\right)\delta V{}_{U}-g^{0}\alpha {}_{N}+\left(L{}^{T}\Delta f_{z}\right){}_{E},\\ \dot{\delta V}{}_{N} & = & -\left(2u{}_{U}+\Omega {}_{U}\right)\delta V{}_{E}+\left(2u{}_{E}+\Omega {}_{E}\right)\delta V{}_{U}+g^{0}\alpha {}_{E}+\left(L{}^{T}\Delta f_{z}\right){}_{N},\\ \dot{\delta V}{}_{U} & = & +\left(2u{}_{N}+\Omega {}_{N}\right)\delta V{}_{E}-\left(2u{}_{E}+\Omega {}_{E}\right)\delta V{}_{N}+\Delta g{}_{U}+\left(L{}^{T}\Delta f_{z}\right){}_{U},\\ \dot{\alpha }{}_{E} & = & -\frac{u{}_{U}+\Omega {}_{U}}{R{}_{E}+h}\Delta r{}_{E}-\frac{\Omega {}_{E}}{R{}_{E}+h}\Delta r{}_{U}-\frac{\delta V{}_{N}}{R{}_{N}+h}+\ldots \\ & \ldots & +\left(u{}_{U}+\Omega {}_{U}\right)\alpha {}_{N}-\left(u{}_{N}+\Omega {}_{N}\right)\beta {}_{U}+\left(L{}^{T}\nu_{z}\right){}_{E},\\ \dot{\alpha }{}_{N} & = & -\frac{u{}_{U}+\Omega {}_{U}}{R{}_{N}+h}\Delta r{}_{N}-\frac{\Omega {}_{N}}{R{}_{N}+h}\Delta r{}_{U}+\frac{\delta V{}_{E}}{R{}_{E}+h}-\ldots \\ & \ldots & -\left(u{}_{U}+\Omega {}_{U}\right)\alpha {}_{E}+\left(u{}_{E}+\Omega {}_{E}\right)\beta {}_{U}+\left(L{}^{T}\nu_{z}\right){}_{N},\\ \dot{\beta }{}_{U} & = & +\frac{u{}_{E}+\Omega {}_{E}}{R{}_{E}+h}\Delta r{}_{E}+\frac{u{}_{N}+\Omega {}_{N}}{R{}_{N}+h}\Delta r{}_{N}+\ldots \\ & \ldots & +\left(u{}_{N}+\Omega {}_{N}\right)\alpha {}_{E}-\left(u{}_{E}+\Omega {}_{E}\right)\alpha {}_{N}+\left(L{}^{T}\nu_{z}\right){}_{U},\\ \dot{\Delta g}{}_{U} & = & 0. \end{array}$$

To make INS equations less cumbersome, we omit primes in all coefficients of the INS errors because the equations still hold true to within linear approximation. In addition, we consider the continuous-time version of a linear dynamic system for the sake of notation’s simplicity.

### 2.4. Measurements and the Estimation Problem

We use a commonly accepted loosely coupled GNSS-INS integration scheme with the feedback into inertial solution, the reason behind being its equivalence with the tightly coupled integration under a sufficient number of GNSS measurements. We assume that the misalignment calibration experiment is carried out in a favorable GNSS environment. In the estimation, we use a GNSS-derived position and velocity solution obtained from Doppler measurements. For them, timing errors play a significant role, so they are described separately below.

#### 2.4.1. Time Synchronization Errors between INS and GNSS

The benefit and methods of estimating the time synchronization errors between INS and GNSS are shown both by numerical simulation of low-cost GNSS-aided INS integration with feedback [19] and by the processing of real data from aircraft flights with a strapdown INS [20]. For our research, we have also performed the numerical simulation, which supports the above results (see Section 3.4).

Let $r_{x}^{\prime }\left(t\right)$ and $V_{x}^{\prime }\left(t\right)$ be the calculated INS position and velocity, respectively, computed at some time *t*. We then define the time synchronization errors $\tau^{pos,\;k}$, $\tau^{vel,\;k}$ for two antennas (k=1,2) between INS and GNSS solutions as follows:

- $r_{x}^{k}\left(t-\tau^{pos,\;k}\right)$ is a GNSS-derived position of the *k*-th GNSS antenna computed at time *t*,
- $V_{x}^{k}\left(t-\tau^{vel,\;k}\right)$ is a GNSS-derived velocity of the *k*-th GNSS antenna computed at time *t*.

We assume magnitudes of time synchronization errors to lie typically within 0.1–0.2 s, i.e., within a few GNSS time steps. Hence, we accept the following relations:

(18) $$\begin{array}{ccc} r_{x}^{k}\left(t-\tau^{pos,\;k}\right) & = & r_{x}^{k}-V_{x}^{k}\tau^{pos,\;k}, \end{array}$$

(19) $$\begin{array}{ccc} V_{x}^{k}\left(t-\tau^{vel,\;k}\right) & = & V_{x}^{k}-{\dot{V}}_{x}^{k}\tau^{vel,\;k}, \end{array}$$

(20) τ˙pos,k=0,.τ˙vel,k=0.

In (18) and (19) and further on, we specify only time instants different from *t*.

#### 2.4.2. Angular Misalignment between Instrumental and Body Frames

In this section, we mathematically formulate the problem of the angular misalignment between INS and dual-antenna GNSS. Let us recall the underlying assumptions:

1. Origins of the instrumental and body frames are the same;
2. Constant lever arms of two GNSS antennas $A^{1}$, $A^{2}$ in the body frame $\ell_{b}^{1}$, $\ell_{b}^{2}$ are known;
3. Instrumental and body frames slightly differ.

The first proposition follows from the definition of the body frame (see Table 1). As for the second assumption, the components $\ell_{b}^{1}$, $\ell_{b}^{2}$ are found or calculated from the technical documentation of the carrier object. If the angular misalignment between reference frames is large, one can deduce its approximate magnitude from the same technical documentation, thus reducing the problem to small angles, as the third assumption states.

From the assumptions above, for ${\bar{\ell }}^{1}$, ${\bar{\ell }}^{2}$ we have

(21) $$\begin{array}{c} \ell_{z}^{1}=\left(I+{\hat{\varkappa }}_{b}\right)\ell_{b}^{1},\;\ell_{z}^{2}=\left(I+{\hat{\varkappa }}_{b}\right)\ell_{b}^{2}, \end{array}$$

i.e., the attitude of the IMU instrumental frame *z* relative to the body frame *b* is determined by Euler rotation vector $\varkappa_{b}=\left(\varkappa_{1},\;\varkappa_{2},\;\varkappa_{3}\right){}^{T}$. The constancy of $\varkappa_{b}$ fully depends on the carrier object being rigid and stiff enough for GNSS antennas and IMU spatial separation to stay the same, so we believe that

(22) $$\begin{array}{c} {\dot{\varkappa }}_{b}=0. \end{array}$$

#### 2.4.3. INS Attitude Errors

The INS error equations given in Section 2.3 imply several reference frames as per Table 2 below.

The origin of true frames *x*, *z* is the IMU reference point *M*. The origin of computed frames $x^{\prime }$, *y*, $z^{\prime }$ is a computed IMU position $M^{\prime }$. Euler rotation vectors $\alpha_{x}^{0}$, $\beta_{x}^{0}$ and $\gamma_{x}^{0}$ represent transformations between close reference frames *x*, $x^{\prime }$, and *y*, so that for the components of any vector *v*, we have the following:

(23) $$\begin{array}{c} v_{z}=Lv_{x},\;v_{z^{\prime }}=L^{\prime }v_{x^{\prime }},\;v_{y}=\left(I+{\hat{\alpha }}_{x}^{0}\right)v_{x},\;v_{y}=\left(I+{\hat{\beta }}_{x}^{0}\right)v_{x^{\prime }},\;v_{x^{\prime }}=\left(I+{\hat{\gamma }}_{x}^{0}\right)v_{x}, \end{array}$$

with the linear relation between Euler rotation vectors $\alpha_{x}^{0}$, $\beta_{x}^{0}$, $\gamma_{x}^{0}$ and INS errors represented as follows:

(24) $$\begin{array}{ccc} \alpha_{x}^{0} & = & \left(\alpha {}_{E},\;\alpha {}_{N},\;\beta {}_{U}+\frac{\tan\varphi }{R{}_{E}+h}\Delta r{}_{E}\right){}^{T}\;\;,\;\;\beta_{x}^{0}=\left(\alpha {}_{E}+\frac{\Delta r{}_{N}}{R{}_{N}+h},\;\alpha {}_{N}-\frac{\Delta r{}_{E}}{R{}_{E}+h},\;\beta {}_{U}\right){}^{T}\;\;, \end{array}$$

(25) $$\begin{array}{ccc} \gamma_{x}^{0} & = & \left(-\frac{\Delta r{}_{N}}{R{}_{N}+h},\;\;\frac{\Delta r{}_{E}}{R{}_{E}+h},\;\;\frac{\tan\varphi }{R{}_{E}+h}\Delta r{}_{E}\right){}^{T}\;\;,\;\alpha_{x}^{0}=\gamma_{x}^{0}+\beta_{x}^{0}. \end{array}$$

Thus, introducing INS attitude errors, we follow the ideology of [18] with slightly different notation. The complexity may seem excessive, but it keeps mathematical rigor in our derivations.

#### 2.4.4. Position Measurements

Let ${\bar{r}}^{k}$ be the radius vector for the *k*-th GNSS antenna (k=1,2), as derived from GNSS pseudoranges [9] and converted to the Earth-centered geodetic navigation frame *x*. The residual position measurement for our linear estimation problem then becomes:

(26) $$\begin{array}{c} \zeta^{pos,\;k}\overset{def}{=}r_{x^{\prime }}^{\prime }-r_{x}^{k}\left(t\;-\;\tau^{pos,\;k}\right)+L^{\prime }{}^{T}\ell_{b}^{k}. \end{array}$$

Adding and subtracting $r_{x^{\prime }}$ from the right part of (26), substituting (18), (21), (24), (25) into (26), and using the relation $r_{x}+\ell_{x}^{k}=r_{x}^{k}$, yield a linearized model for the residual position measurement at the GNSS epoch *t* for the *k*-th antenna:

(27) $$\begin{array}{c} \zeta^{pos,\;k}=\Delta r_{x}-\hat{L^{\prime }{}^{T}\ell_{b}^{k}}\beta_{x}^{0}-{\hat{r}}_{x}^{k}\left(t\;-\;\tau^{pos,\;k}\right)\gamma_{x}^{0}+V_{x}^{k}\left(t\;-\;\tau^{vel,\;k}\right)\tau^{pos,\;k}+L^{\prime }{}^{T}{\hat{\ell }}_{b}\varkappa_{b}+\rho^{pos,\;k}, \end{array}$$

where $\rho^{pos,\;k}$ is a stochastic error with a priori known moments.

#### 2.4.5. Velocity Measurements

Let $\Theta_{z}$ be the angular velocity of the frame Mz relative to the Earth so that $\Theta_{z}=\omega_{z}-Lu_{x}$. Having the GNSS velocity solution derived from Doppler measurements [21], we introduce a residual velocity measurement:

(28) $$\begin{array}{c} \zeta^{vel,\;k}\overset{def}{=}V_{x^{\prime }}^{\prime }-V_{x}^{k}\left(t\;-\;\tau^{vel,\;k}\right)+L^{\prime }{}^{T}{\hat{\ell }}_{b}^{k}\Theta_{z}^{\prime }. \end{array}$$

Similarly to the derivation of (27), we add and subtract $V_{x}$ from the right part of (28), substitute (19), (21), (24) into (28), and use the relation for linear velocities of two points ($A^{k}$ and *M*) of a rigid body $V_{x}^{k}=V_{x}-L{}^{T}{\hat{\Theta }}_{z}\ell_{z}$. The linearized model for the residual velocity measurement at GNSS epoch *t* for the *k*-th antenna then becomes:

(29) $$\begin{array}{ccc} \zeta^{vel,\;k} & = & \delta V_{x}-\left({\hat{V}}_{x^{\prime }}^{\prime }+\hat{L^{\prime }{}^{T}{\hat{\ell }}_{b}^{k}\Theta_{z}^{\prime }}\right)\alpha_{x}^{0}-L^{\prime }{}^{T}{\hat{\ell }}_{b}^{k}L^{\prime }{\hat{u}}_{x}^{\prime }\beta_{x}^{0}-L^{\prime }{}^{T}{\hat{\ell }}_{b}^{k}\nu_{z}^{0}+\ldots \\ & \ldots & +{\dot{V}}_{x}^{k}\tau^{vel,\;k}-L^{\prime }{}^{T}{\hat{\Theta }}_{z}^{\prime }{\hat{\ell }}_{b}^{k}\varkappa_{b}+\rho^{vel,\;k}, \end{array}$$

where $\rho^{vel,\;k}$ contains both GNSS measurement noise and the gyroscope stochastic term $\nu_{z}^{s}$, whose moments are known. The coefficient of $\tau^{vel,\;k}$ in (29), taking (2) into account, can be expressed up to linear terms as

(30) $$\begin{array}{c} {\dot{V}}_{x}^{k}=L^{\prime }{}^{T}f_{z}^{\prime }+g_{x^{\prime }}^{\prime }+\left({\hat{\Omega }}_{x^{\prime }}^{\prime }+2{\hat{u}}_{x^{\prime }}^{\prime }\right)V_{x^{\prime }}^{\prime }+L^{\prime }{}^{T}\left({\hat{\Theta }}_{z}^{\prime \;2}-{\hat{\dot{\Theta }}}_{z}^{\prime }\right)\ell_{b}^{k}-{\hat{\Omega }}_{x^{\prime }}^{\prime }L^{\prime }{}^{T}{\hat{\Theta }}_{z}^{\prime }\ell_{b}^{k}. \end{array}$$

Note that (30) may be further simplified if the IMU is stationary, so that $V_{x}\approx \Omega_{x}\approx 0$.

#### 2.4.6. Estimation Problem

Thus, we have reduced the problem of calibration of angular misalignment between dual-antenna GNSS and IMU to a linear stochastic estimation problem with the following 23-dimensional state space vector:

(31) $$\begin{array}{c} \left(\Delta r_{x}{}^{T},\delta V_{x}{}^{T},\alpha {}_{E},\alpha {}_{N},\beta {}_{U},\Delta g{}_{U},\Delta f_{z}^{0}{}^{T},\nu_{z}^{0}{}^{T},\tau^{pos,\;1},\tau^{pos,\;2},\tau^{vel,\;1},\tau^{vel,\;2},\varkappa_{b}{}^{T}\right){}^{T}\;\;. \end{array}$$

The dynamic model for (31) consists of (16), (17), (20), and (22). Equations (27) and (29) form the measurement model. For processing, we use the discrete-time equivalent of these equations. The initial estimate of the state space vector is zero, and the initial covariance matrix of the estimation error is known a priori.

There are many methods based on the Kalman filtering approach [22], which provide estimates for the state vector components (31) either in real time or in post-processing. We use the Potter square root filter version based on Cholesky covariance factorization [23].

## 3. Numerical Simulation

### 3.1. Motivation

After the calibration has been formulated as an estimation problem in the above Section 2.4, we need to ensure its good estimability properties in terms of converging error covariance [24]. As it is usually the case in INS sensor fusion algorithms, its properties mostly depend on the motion of the IMU. Time-varying coefficients $\omega_{z}\left(t\right)$, $f_{z}\left(t\right)$, and L(t) in (27) and (29) define how well components of the state vector will separate from each other in the estimation process, or, in other words, how differently these components will manifest themselves in INS errors. For higher-order time-varying systems such as that being under consideration, predicting its properties analytically from the equations alone is hardly a solvable task in general. In fact, only numerical analysis may provide practical insights for most systems of this kind. Therefore, testing a range of scenarios for the calibration experiment, and choosing the right one to be actually executed, become primary reasons to perform numerical simulation. The secondary reason being mere verification of the consistency between our models and algorithms is also important to support future conclusions.

In our case, it was the simulation that has forced us to take into account effects which a priori seemed quite neglectable even to experts in the field. All in all, we have decided to describe the numerical simulation as an inseparable part of our research.

### 3.2. Inertial Sensors

As a matter of fact, rotational motion is crucial to calibrating angular misalignment. To simulate such motion, we have developed a virtual three-axis turntable. Each axis, being controlled and simulated individually, can perform a number of commands. Their list includes moving into an arbitrary pre-selected position, uniform rotation at a given rate, harmonic oscillations and stopping the rotation.

For each axis of our virtual turntable, according to a pre-defined set of specific commands, we create a twice continuously differentiable analytical function, representing the angle of rotation around this particular axis over time. Combining rotations for three axes then allows us to simulate a wide range of complex motion patterns and calibration scenarios. Using analytic functions for rotation angles, we derive the absolute angular rate $\omega_{z}\left(t\right)$ and the specific force $f_{z}\left(t\right)$ vectors as projected onto the IMU instrumental reference frame. We then calculate their components on a discrete time grid at a high rate. Downsampling to a desired IMU sampling rate of, say, 250 Hz, using arithmetic average, completes the simulation process.

The higher internal frequency of the simulation allows us to properly reproduce the integration (or averaging) which occurs in real inertial sensors. Internal frequency may be set as high as it is required for a given rotation pattern. In practice, one should try larger and larger values until the change in navigation solution becomes negligible. In our case, for a 250 Hz IMU sampling rate, a 256 times higher simulation frequency of 64 kHz has happened to be enough.

For now, the IMU reference point *M* remains stationary in our simulation. Inertial sensor errors satisfy the model (15), being added when appropriate.

### 3.3. GNSS Measurements

Having the IMU rotation ready, so that we may assume angular rate vector components $\omega_{z}\left(t_{i}\right)$ and attitude matrix $L(t_{i})$ to be known at a discrete time grid, the GNSS position and velocity of two antennas $A^{1}$ and $A^{2}$ need to be simulated (see Figure 2). For them, each lever arm vector $\ell_{b}^{1}$ and $\ell_{b}^{2}$ is known component-wise in some carrier body reference frame *b*. Angular misalignment angles $\varkappa_{1}$, $\varkappa_{2}$ and $\varkappa_{3}$ define an Euler rotation vector ϰ with the corresponding rotation matrix *D* according to (11):

(32) $$D=I+\frac{\sin∥\varkappa_{b}∥}{∥\varkappa_{b}∥}{\hat{\varkappa }}_{b}+\frac{1-\cos∥\varkappa_{b}∥}{∥\varkappa_{b}{∥}^{2}}{\hat{\varkappa }}_{b}^{2}.$$

Using the above matrix, the antenna coordinates in geodetic Cartesian axes become

(33) $$A^{k}=M+L{}^{T}D\ell_{b}^{k},$$

with, longitude, latitude and altitude as well as radius vector appropriately calculated.

Velocity vector derivation uses Euler’s rotation formula, so that given the IMU does not perform linear motion, for each antenna, we have

(34) $$V_{x}^{k}=-L{}^{T}{\hat{\Theta }}_{z}D\ell_{b}^{k},\;\Theta_{z}=\omega_{z}-Lu_{x},$$

where the instant rotation rate components $\omega_{z}$ are either taken directly from the simulation of inertial sensors before averaging or derived from the gyroscope output similar to (10).

When necessary, we add GNSS solution errors to the antenna position and velocity as well. The GNSS position stochastic errors, although having a rather complicated nature in practice, happen to have a minor effect on the estimation of angular misalignment. For GNSS velocity derived from Doppler measurements, their stochastic errors appear to be quite close to white noise.

### 3.4. Simulation Results

To demonstrate the rationale behind certain decisions accepted in our calibration method, we have simulated the following effects listed in Table 3 below.

Before proceeding to choose the class of rotational motion for the calibration, one should note that in measurement model (27), (29) the coefficients of the desired $\varkappa_{b}$ parameters contain constant factors ${\hat{\ell }}_{b}^{k}$, and for them, we have $\mathrm{rank}\;\left({\hat{\ell }}_{b}^{1}\;\;{\hat{\ell }}_{b}^{2}\right)\leq 3$, so it can be potentially less than the dimension of $\varkappa_{b}$. Therefore, the choice of two GNSS antenna locations ($\ell_{b}^{1}$, $\ell_{b}^{2}$) with respect to the IMU has a direct impact on the estimability of all three parameters $\varkappa_{1}$, $\varkappa_{2}$, $\varkappa_{3}$. The necessary conditions for them to be estimable is $\ell_{b}^{1}\neq c\ell_{b}^{2}$ for any non-zero constant *c*. In the simulation, this condition is satisfied at all times, unlike the next argument in Section 4 dedicated to real data processing, where the actual sensor setup did not allow for that.

For the misalignment calibration problem, we suggest using so-called *conical* motions. They comprise simultaneous rotations around two perpendicular axes. For them to be carried out on a turntable, two of its axes perform harmonic oscillations out of phase by a quarter of a full period with each other. The term *conical* arises from one of the instrumental axes moving along the generatrix of a certain circular or elliptical cone (depending on amplitudes of the above harmonic oscillations).

**Figure 3:**
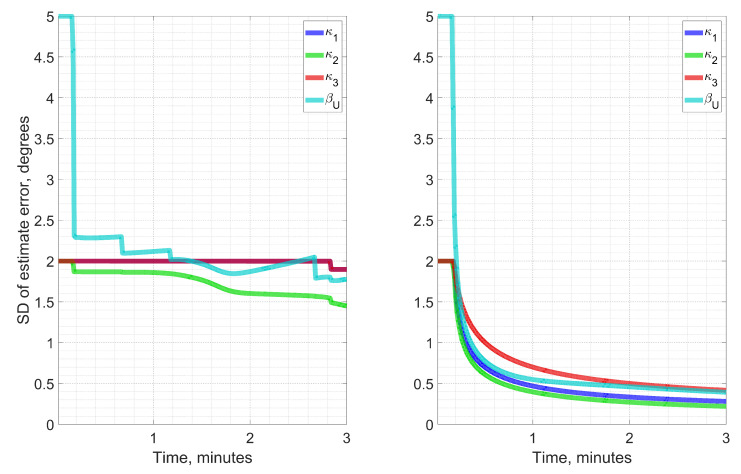
Estimated standard deviations (SD) for $\varkappa_{b}$, $\beta {}_{U}$ in two types of rotation: maytagging-like (**left**), and conical (**right**), showing the advantage of the latter one with steady convergence to lower SD values for all components.

Figure 3 demonstrates the difference in estimation process for two different rotation types in terms of $\varkappa_{b}$. The first type of motion (see the left inset) is a sequence of four rotations by 90 around vertical axis $x_{3}$, which approximately coincides with the second instrumental one $z_{2}$. After each rotation by 90, there is a static position. Axes $z_{1}$ and $z_{3}$ lie near the horizontal plane $x_{1}x_{2}$. As a result, two GNSS antennas move along horizontal circles. Rotations such as these, so that the IMU has different heading angles with a roughly 90 increment, are similar to maytagging—a conventional technique used for gyrocompassing using low-grade inertial sensors [2]. The second type of rotation (see the right inset of Figure 3) is the conical motion described above. The figure clearly shows that conical rotation provides a strictly monotonic decrease of the estimate error covariance converging to zero over time, while the maytagging does not do so.

**Figure 4:**
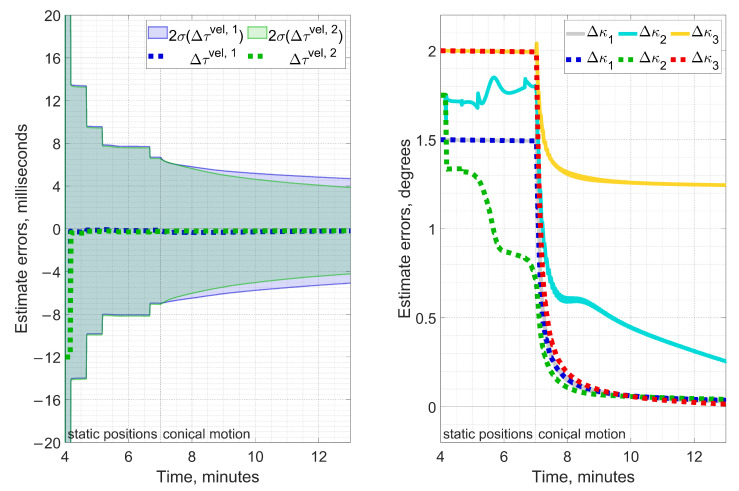
The left plot shows errors of $\tau^{vel,\;1}$, $\tau^{vel,\;2}$ estimates (dashed lines) and their corresponding 2-σ intervals (semitransparent). The right plot contains errors of $\varkappa_{b}$ estimates in two cases: with GNSS-derived velocity delays $\tau^{vel,k}=12\;\mathrm{msec}$ included in the estimation problem (dashed lines) and without them (solid lines). The latter have an $\varkappa_{3}$ estimate converging to a wrong value and an $\varkappa_{2}$ error decreasing much slower than it should. For a smaller magnitude of $\tau^{vel,k}$, the estimation errors still may remain significant.

The second point which the numerical simulation has given us an insight into refers to the timing errors between inertial and GNSS information while performing the angular misalignment calibration. Although it is almost self-evident that these should be taken into consideration, the actual figures have become a surprise even for experienced engineers working in the field. Figure 4 indicates that even 12-millisecond timing errors $\tau^{vel,\;k}$, (k=1,2), which correspond to only 3 IMU samples at 250 Hz, can be critical. The dashed lines stand for the errors in estimating $\tau^{vel,\;k}$ (left plot) and $\varkappa_{b}$ (right plot) when calibration models include the timing skew. Solid lines represent the case when $\tau^{vel,\;k}$ is omitted under simulated 12-millisecond delay in GNSS measurements. It apparently becomes an issue for estimating $\varkappa_{2}$, $\varkappa_{3}$, with their estimates swaying away from reference values. In addition to Figure 3, the plots below confirm that once the conical rotation starts, the estimated misalignment errors immediately begin to converge. Timing errors $\tau^{vel,\;k}$ appear to have good estimability right away from the very first rotation.

From the same assessment, it follows that even phase delays of a fraction of the inertial time step in angular rate measurements should be accounted for in both simulation (34) and estimation (30), since they produce significant calibration errors, albeit not as large as in the example that Figure 4 illustrates.

**Figure 5:**
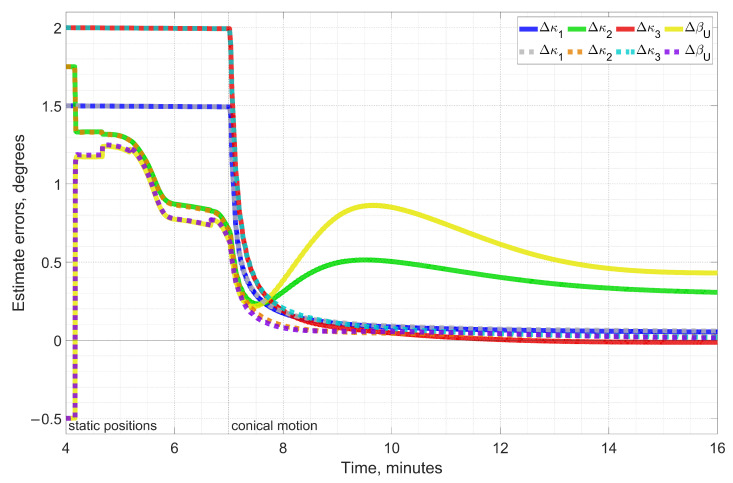
Errors of $\varkappa_{b}$, $\beta {}_{U}$ estimates using (**a**) conventional Euler attitude integration (solid lines), and (**b**) our algorithm described in Section 2.1.2 (dashed lines). Replacing the attitude integration algorithm alone changes the convergence from non-existing to very good.

Another issue that the numerical simulation has revealed appeared to be a substantial difference between attitude integration methods. Although simpler integration methods are usually considered sufficient for MEMS gyroscopes, Figure 5 indicates that replacing the method alone changes the estimation error completely once conical rotation starts. For conventional Euler integration, which accounts only for the first term in the Bortz kinematic Equation (9), estimation errors of $\varkappa_{2}$ and $\beta {}_{U}$ shown in green and yellow, respectively, do not converge to zero over time. We believe that this is due to the fact that a more accurate attitude integration algorithm provides errors much more closely conforming to the INS error Equation (15) for their systematic parts.

In this section, we deliberately do not show any results with sensor errors containing stochastic terms, because qualitative analysis does not depend on them. In fact, such simulations with Gaussian noises were carried out as well to test our processing software. Overall, our experience has shown that estimation problems of this kind are barely solvable in practice without proper simulation.

## 4. Experimental Results

**Figure 6:**
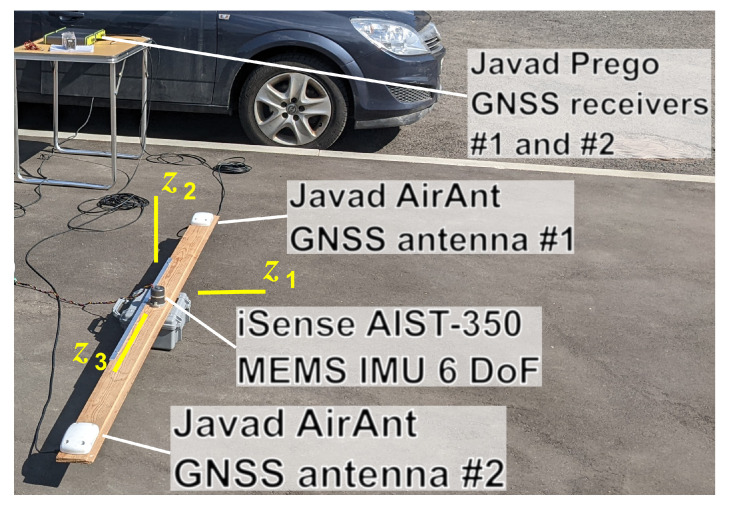
An experimental setup used for preliminary validation of the calibration method. Two GNSS antennas and a 6 DoF MEMS IMU are fixed to a single base plank. After initial alignment, the whole structure undergoes series of rotations of different types performed by hand.

For preliminary validation, two similar calibration experiments have been performed using the setup shown in Figure 6.

In these experiments, we used high-precision GNSS equipment, namely Javad™ Prego^®^ receivers and AirAnt^®^ antennas. For inertial sensors, we took an iSense™ AIST-350 thermally stabilized MEMS IMU based on LPY510 gyroscopes and ADXL326 accelerometers by ST Microelectronics™ and Analog Devices™, respectively. GNSS receivers were operating at 10 Hz, while IMU records had a 250 Hz sampling rate. Both experiments, after the 4 min initial alignment phase, included three types of rotation for approximately 6 min each, with 4 min static intervals between them:

1. Oscillations around the roll axis (referred to as γ(t) oscillations below) with amplitude up to 10 and nearly constant yaw ψ(t) and pitch θ(t) angles;
2. Oscillations around the yaw axis (referred to as ψ(t) oscillations below) with amplitude around 15 with nearly constant roll γ(t) and pitch θ(t) angles, both close to zero;
3. Conical rotations, in which IMU performed minor linear motion, and GNSS antennas were traveling along circles in anti-phase with each other, antenna lever arm vectors sweeping along generatrix lines with 40–50 aperture, at a period of around 2–4 s. These motions may be considered as a composition of the previous two.

All rotations were performed manually. Lever arms for both antennas had their lengths around 0.8 m. Note that in our experiment, lever arm vectors ${\bar{\ell }}^{1}$ and ${\bar{\ell }}^{2}$ happen to be collinear, so that the IMU reference point *M* lies on their baseline $A^{1}A^{2}$. Hence, parameter $\varkappa_{3}$ is not estimable under the given geometry. From this point on, we consider the estimability of only two parameters $\varkappa_{1}$ and $\varkappa_{2}$ for the angular misalignment between IMU and dual-antenna GNSS reference frames. Their true values, as described in the Introduction, remain unknown, and there exist no reasonable means of measuring them directly. However, since between the two experiments, our instrumental setup has not changed, we expect estimates for $\varkappa_{1}$ and $\varkappa_{2}$ to repeat.

**Figure 7:**
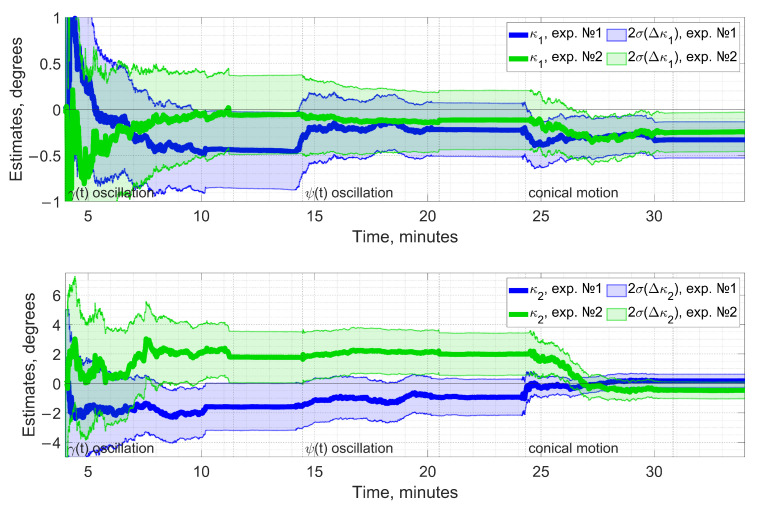
Estimates for $\varkappa_{1}$ (**top**), $\varkappa_{2}$ (**bottom**) and their corresponding 2-σ confidence intervals (semitransparent) in two similar experiments. The intervals eventually overlap with a desired sub-degree level of precision, indicating consistent results.

Figure 7 illustrates the estimation process over time for both experiments throughout different types of rotation. Estimates for $\varkappa_{1}$ and $\varkappa_{2}$ appear as solid lines along with their predicted 2-σ confidence intervals. The latter seem to well overlap by the end of the calibration, indicating that the estimates are consistent with each other in two experiments at a sub-degree precision. Experimental data used in this Section are available in Supplementary Materials for processing.

Performing the first two types of rotation in our experiment was motivated by the potential possibility of replacing conical motion, being more difficult to implement in practice, with two of its constituents, namely rotations around each axis individually. To address this, let us refer to Table 4 with Figure 7 serving for further clarification.

The estimated error covariance for $\varkappa_{1}$, $\varkappa_{2}$ noticeably decreasing after the execution of conical motion implies that rotations around roll and yaw axes separately do not provide proper convergence. So, from the covariance analysis, we recognize that conical rotation is the preferred type of motion ensuring estimability for the angular misalignment calibration in real experiments and further supporting the results of numerical simulation.

## 5. Discussion

We have reduced the problem of angular misalignment calibration between the instrumental reference frame associated with an IMU, and the carrier body reference frame with known locations of two GNSS antennas in it, to a conventional linear stochastic estimation problem. The research is relevant to all applications aimed at tracking orientation using a low-grade IMU and dual-antenna GNSS within a sub-degree level of precision. Appropriate measurement models have been derived to extend conventional loosely coupled GNSS/INS sensor fusion filtering for including parameters required for the misalignment calibration. It uses a GNSS position solution and velocity derived from Doppler observables. Since the algorithm has inertial sensor biases in its state vector subject to estimation, it is expected to be immune to run-to-run bias change inherent to most lower-grade inertial sensors. Presumably, after compensating for the initial misalignment, the estimation may continue running in the background to account for slower structural deformations over time. This, however, requires additional research for confirmation.

The numerical simulation in Section 3.4 has shown that:

1. The preferred motion pattern for calibrating angular misalignment includes conical rotation;
2. The following key issues appeared to be essential to successful estimation:
  - Taking the time synchronization error between IMU and GNSS data into account at the few-millisecond-level;
  - The above includes phase delay inherent to integrating (or averaging) gyroscopes;
  - Modifying the attitude integration algorithm to produce errors properly obeying the INS error equations.

The processing of real experimental data has shown the feasibility of the proposed calibration method, and it produced consistent results in agreement with the numerical simulation. The final predicted standard deviation of the misalignment error does not exceed 0.25.

However, more extensive validation is planned for the future, since the real misalignment angles, i.e., the “ground truth”, seem to be practically unavailable in real applications that use MEMS sensors. The validation may be achieved through a variety of approaches, the first being increasing the number of experiments to a statistically significant amount. The second option is to perform the calibration using a high-grade inertial sensors. A navigation-grade INS is able to produce attitude angles autonomously, so that its instrumental frame may be directly aligned to GNSS antennas within some few arc minutes. After that, MEMS gyroscope and accelerometer data may be used to simulate low-grade IMU output for our calibration algorithm to be applied to. The third approach is an indirect one, the idea behind being to show that the navigation solution becomes more accurate after compensating for the estimated misalignment angles. Having its own importance in itself, this approach will become our primary focus for future research.

In this work, we used the GNSS-derived position and velocity obtained from code pseudoranges and Doppler measurements, respectively, since there exists GNSS equipment not able to record phase measurements. The first two measurement types seem to be available for an external processing in a wider range of GNSS devices rather than phase measurements. For many general applications, lower-accuracy GNSS sensors are preferable due to their smaller cost and size. Still, using phase measurements, and developing the corresponding extension to the estimation problem, including INS-aided carrier phase ambiguity resolution on-the-fly (i.e., deeply-coupled GNSS/INS for MEMS) is the next possible development branch in our research.

## Figures and Tables

**Table 1:** General notation.

Symbol	Meaning
$A^{1},\;A^{2},\;M$	GNSS antenna locations and IMU reference point, respectively
${\bar{\ell }}^{1},\;{\bar{\ell }}^{2}$	antenna lever arm vectors, i.e., ${\bar{\ell }}^{k}={\bar{MA}}^{k}$
$b_{1},\;b_{2},\;b_{3}$	carrier body reference frame: $b_{1}$ — roll axis, $b_{2}$ — normal axis, $b_{3}$ — transverse axis
$z_{1},\;z_{2},\;z_{3}$	respective IMU instrumental axes
$\varkappa_{1},\;\varkappa_{2},\;\varkappa_{3}$	components of the Euler rotation vector transforming from Mb frame to Mz
$x_{1},\;x_{2},\;x_{3}$	navigation frame: $x_{1}$ — eastward (E), $x_{2}$ — northward (N), $x_{3}$ — upward (U)
*L*	IMU attitude matrix, transforming from Mx frame to Mz
γ,θ,ψ	corresponding roll, pitch and true heading angles, respectively
$\omega_{z},\;f_{z}$	angular rate and specific force vectors as projected onto the instrumental axes
$\omega_{z}^{\prime },\;f_{z}^{\prime }$	their components as measured by respective inertial sensors
$\bar{g},\;\bar{u}$	the Earth’s local gravity and rotation velocity vectors
φ,λ,h	geodetic coordinates (latitude, longitude, altitude)
$\bar{V}$	IMU velocity vector
*t*	time

**Table 2:** Reference frames.

Axes	Description
$x_{1},\;x_{2},\;x_{3}$	true navigation frame
$z_{1},\;z_{2},\;z_{3}$	true IMU instrumental frame
$x_{1}^{\prime },\;x_{2}^{\prime },\;x_{3}^{\prime }$	computed navigation frame as a result of INS coordinate errors
$z_{1}^{\prime },\;z_{2}^{\prime },\;z_{3}^{\prime }$	computed instrumental frame as result of applying operator $L^{\prime }$ to $x^{\prime }$ axes
$y_{1},\;y_{2},\;y_{3}$	computed navigation frame as result of applying operator $L^{\prime }{}^{T}$ to *z* axes

**Table 3:** Simulated effects.

Description	Parameters	Typical Values
Different spatial configurations of sensor setup	$\ell_{b}^{k}$	∼1 m,
Angular misalignment between *b* and *z* frames	$\varkappa_{b}$	∼3°
Initial alignment errors	$\Delta \psi \left(t_{0}\right)=\beta {}_{U}$	∼1°
Inertial sensor errors	$\Delta f_{z}$, $\nu_{z}$	∼1 cm/s^2^, 10°/h
GNSS velocity solution bias		1 cm/s
Time delays of GNSS solution	$\tau^{pos,\;k}$, $\tau^{vel,\;k}$	∼10 ms

**Table 4:** Estimation results prior and after the conical rotation.

Numerical Values	$\varkappa_{1}$ (exp. №1/2)	$\varkappa_{2}$ (exp. №1/2)
1-σ before conical motion	0.15°/0.16°	0.61°/0.72°
1-σ after conical motion	0.10°/0.11°	0.23°/0.28°
Estimate before conical motion	−0.23°/−0.12°	−0.93°/+2.00°
Estimate after conical motion	−0.33°/−0.25°	+0.16°/−0.24°

## Data Availability

MEMS IMU and GNSS measurements in RINEXv3.04 format are available in Supplementary Materials along with a description of data and the experiments. Please refer to the **readme.txt** file in the archive for details.

## Supplementary Materials

The following supporting information can be downloaded at: https://www.mdpi.com/article/10.3390/s23010077/s1, Archive **imu.zip**: IMU gyroscopes and accelerometers data; Archive **gnss.zip**: dual-antenna GNSS data in RINEXv3.04 format; Photo **setup-overview.jpg**: overview of the experimental setup; Photo **setup-geometry.png**: scheme of setup geometry; Text file **experiment-info.md**: detailed description of experiment.

## Abbreviations

The following abbreviations are used in this manuscript:

IMU	Inertial Measurement Unit
INS	Inertial Navigation System
GNSS	Global Navigation Satellite System
MEMS	Microelectromechanical Sensor
SD	Standard Deviation
RTK	Real-Time Kinematic
DoF	Degree of Freedom
