# Supplementary figures and images for: Angular Misalignment Calibration for Dual-Antenna GNSS/IMU Navigation Sensor
Source: Sensors (Basel). 2022 Dec 21;23(1):77. doi: 10.3390/s23010077 (PMC9824128; doi:10.3390/s23010077)

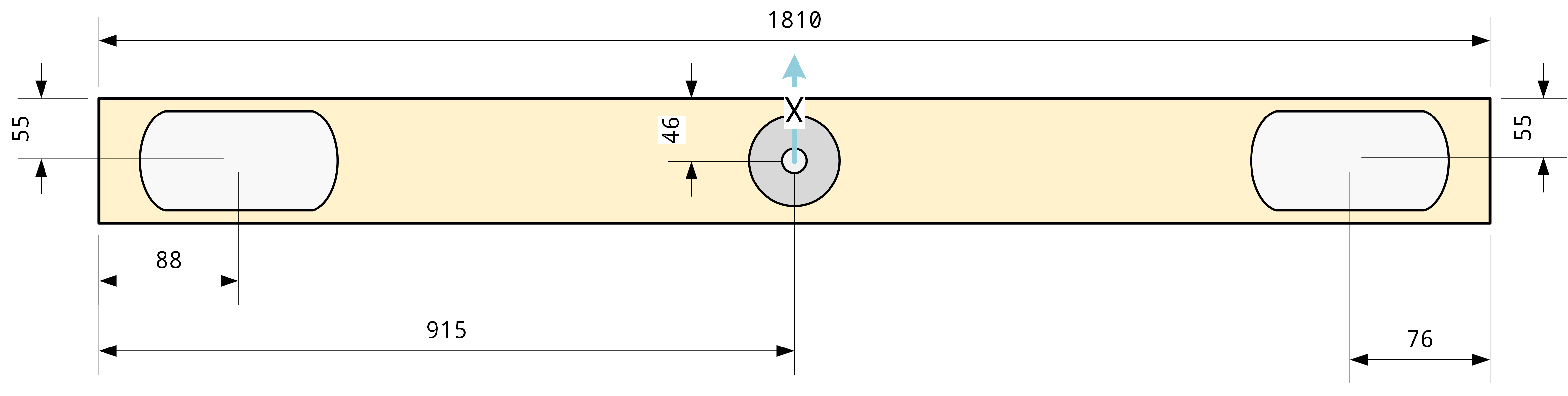

Supplement: Supplementary file 1 [file sensors-23-00077-s001.zip › info/setup-geometry.png]

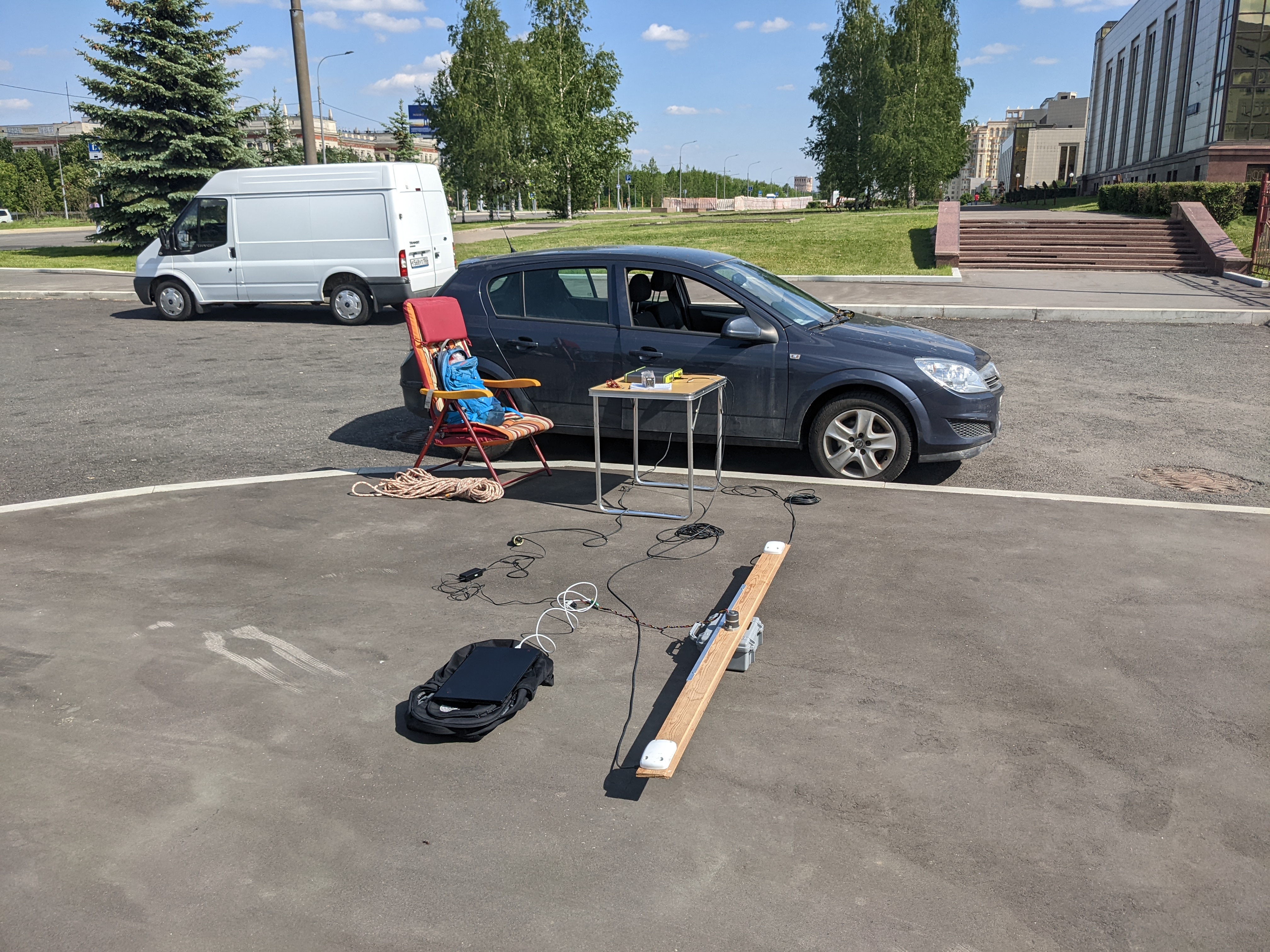

Supplement: Supplementary file 1 [file sensors-23-00077-s001.zip › info/setup-overview.jpg]
